# The Strategy of Picornavirus Evading Host Antiviral Responses: Non-structural Proteins Suppress the Production of IFNs

**DOI:** 10.3389/fmicb.2018.02943

**Published:** 2018-12-11

**Authors:** Yining Wang, Lina Ma, Laszlo Stipkovits, Susan Szathmary, Xuerui Li, Yongsheng Liu

**Affiliations:** ^1^State Key Laboratory of Veterinary Etiological Biology, Lanzhou Veterinary Research Institute, Chinese Academy of Agricultural Sciences, Lanzhou, China; ^2^RT-Europe Research Center Ltd., Budapest, Hungary

**Keywords:** IFNs, picornaviruses, non-structural proteins, immune evasion, signaling pathways

## Abstract

Viral infections trigger the innate immune system to produce interferons (IFNs), which play important role in host antiviral responses. Co-evolution of viruses with their hosts has favored development of various strategies to evade the effects of IFNs, enabling viruses to survive inside host cells. One such strategy involves inhibition of IFN signaling pathways by non-structural proteins. In this review, we provide a brief overview of host signaling pathways inducing IFN production and their suppression by picornavirus non-structural proteins. Using this strategy, picornaviruses can evade the host immune response and replicate inside host cells.

## Introduction

**FIGURE 1:**
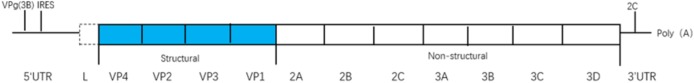
The viral genome is a single-stranded(ss) RNA, encoding a single open reading frame (ORF), an untranslated region (UTR) at either terminus, and a poly(A) tail at the 3′ end. The ORF is translated as a polyprotein, which is processed by viral proteases to release the structural proteins (VP1-4) needed to assemble virus capsids, and the non-structural proteins (2A-2B-2C-3A-3B-3C-3D pro and in some genera Lpro).

Picornaviruses are small, non-enveloped, positive-strand RNA viruses that infect diverse animal and human hosts (Ehrenfeld et al., 2010; Feng et al., 2014b). As one of the largest viral families, picornaviruses contain 31 genera and 54 species, including cardioviruses [e.g., *encephalomyocarditis virus* (EMCV) and *Theiler’s virus* (TEV)], enteroviruses [e.g., *enterovirus* 71 (EV71); *poliovirus* (PV); coxsackievirus (CV); and rhinovirus (RV)], hepatitis A virus (HAV), and foot-and-mouth disease virus (FMDV) (Feng et al., 2014b). Picornavirus genomes are single-stranded RNAs (7,000 to 9,000 nucleotides in length) which consist (from 5′ to 3′) of a 5′ untranslated region (UTR), a single open-reading frame (ORF), a 3′ UTR, and a poly(A) tail (Figure 1; Feng et al., 2014b). The ORF is translated into a polyprotein, which is processed by viral proteases into structural proteins (VP1–VP4) and non-structural proteins (2A, 2B, 2C, 3A, 3B, 3C, and 3D pro, and in some genera, L pro). Structural proteins are used to assemble viral capsids whereas non-structural proteins replicate the genomic RNA in conjunction with cell proteins (Argos et al., 1984; Buenz and Howe, 2006; Ehrenfeld et al., 2010).

Interferons (IFNs) which play important roles in regulation and activation of host immune responses, were first discovered by Isaacs and Lindenmann in 1950s (Isaacs and Lindenmann, 2015; Klotz et al., 2017). IFNs are classified into three categories according to their antiviral activities, genetic, structural and functional features and their cognate receptors (Nagano and Kojima, 1954): type I (IFN-α, IFN-β, IFN-δ, IFN-𝜀, IFN-ζ, IFN-κ, IFN-τ, and IFN-ω), type II (IFN-γ) (Klotz et al., 2017), and type III (IFN-λ1 or IL-29, IFNλ-2 or IL-28A, IFNλ-3 or IL-28B, and IFN-λ4) (Schroder et al., 2004; Gonzáleznavajas et al., 2012). Type I IFNs typically have antiviral effects and are the most broadly expressed, well-known antiviral IFNs. Although type I IFNs can be secreted by most parenchymal cells, the main type I IFN producer is plasmacytoid dendritic cell (pDC) (Coccia and Battistini, 2015; Kindler et al., 2016). Type II IFN is produced by activated T cells and NK cells and predominantly induce macrophage activation stimulating their activity against ingested intracellular non-viral pathogens (Coccia and Battistini, 2015). Type III IFNs are produced by epithelial cells, leukocytes, intestinal eosinophils and pDCs (Ank et al., 2006; Hillyer et al., 2012; Raki et al., 2013; Hernandez et al., 2015; Mahlakoiv et al., 2015; Pervolaraki et al., 2017). Type III IFNs are similar to type I IFNs, and also play roles in regulating the host antiviral response (Reid and Charleston, 2014; Kindler et al., 2016).

Viruses develop various strategies to inhibit secretion of IFNs and promote viral replication inside host cells. Mounting evidence shows that infecting viruses can evade IFN response either by suppressing IFN production or by blocking IFN induction of interferon-stimulated gene factors (ISGs) (Zinzula and Tramontano, 2013; Fensterl et al., 2015). Viral non-structural proteases play an important role in this process. In this review, we summarize our current knowledge of the role of picornavirus non-structural proteases in antagonizing IFN induction via different signaling pathways to inhibit host antiviral responses.

## Signaling Pathways Inducing IFN Production

**FIGURE 2:**
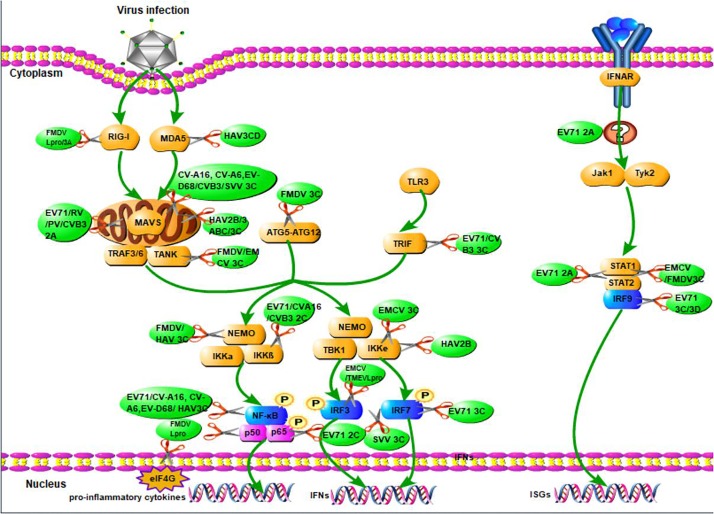
The overview of picornavirus non-structural proteins suppress the production of IFNs. When viruses infect organisms, the host innate immune system detects the presence of pathogen-associated molecular patterns via host pattern recognition receptors, then recruit a number of specific adaptor proteins to trigger a signaling cascade leading to the establishment of an antiviral state based on IFN and proinflammatory cytokines induction. The green arrays represent observations that have yet to be associated with a specific mechanism of action of the production of IFNs. The scissors illustrate the inhibition of viral non-structural proteins in the production of IFNs.

When viruses infect organisms, the host innate immune system detects the presence of pathogen-associated molecular patterns via host pattern recognition receptors (PRRs) (Vaccari et al., 2014; Coccia and Battistini, 2015). These include transmembrane PRRs such as Toll-like receptors (TLRs), cytosolic RIG-like RNA helicases such as melanoma differentiation-associated gene (MDA-5), retinoic acid induced gene-I (RIG-I), and other molecules (Barbé et al., 2014; Wu and Chen, 2014). PRRs recruit a number of specific adaptor proteins to trigger a downstream signaling cascade and activate three major pathways to produce IFNs: the nuclear factor kappa-light-chain-enhancer of activated B cells (NF-κB) (Coccia and Battistini, 2015), the mitogen-activated protein kinase (MAPK), and the IFN regulatory factor (IRF) pathways (Akira et al., 2006; Honda and Taniguchi, 2006). IFNs can signal in an autocrine or paracrine manner to induce hundreds of ISGs that fortify host defenses (Figure 2; Pham et al., 2016).

Induced IFNs exert their activity by binding and activating IFN receptors. Type I IFN receptors are composed of interferon-α/β receptors 1 and 2 subunits (IFNAR1 and IFNAR2) (Olière et al., 2011). Type III IFNs receptors are composed of two different chains, the IFN-λR1 high-affinity chain and IL-10R2 low-affinity chain (Chasset and Arnaud, 2017). The binding of type I IFNs with its receptor leads to the phosphorylation of Janus kinase1 (JAK1) and tyrosine kinase 2 (TYK2). These activated kinases then phosphorylate STAT1 and STAT2 and promote STAT1 and STAT2 heterodimerization. The resulting heterodimer interacts with IRF9 to form a heterotrimeric complex called interferon-stimulated gene factor 3 (ISGF3) (Peng et al., 2017). This complex then translocate to the nucleus and binds a 12–15 bp IFN-α/β-stimulated response element (ISRE’ 5′-G/ANGλN2GλCT-3′), which regulates the transcription of over 300 IFN-stimulated genes, some of which lead to IFN-α and IFN-β expression (Figure 2). Type III IFNs have almost the same process with that of type I IFNs (Blaszczyk et al., 2016; Chasset and Arnaud, 2017).

Type II IFN receptor is composed of two subunits, IFNGR1 and IFNGR2. After the binding of IFN-γ proteins to IFNGR, STAT1 homodimers is formed and bind to IFN-γ-activated site (GAS) enhancer elements with the help of the promoters of IFN-stimulated genes resulting in the production of genes encoding pro-inflammatory cytokines and apoptotic factors. Furthermore, IFN-γ can also activate STAT3 homodimers, which result in the production of pro-inflammatory cytokines and anti-inflammatory cytokines IL-10 (Gonzáleznavajas et al., 2012).

In addition, macrophage recognition of virions induces the secretion of IL-12, IL-18 and other cytokines (Platanias, 2005; Capobianchi et al., 2015). IL-12 binds to the receptor complex consisting of IL12Rβ1 and IL12Rβ2, stimulating NK cells and instigating a signaling cascade that induces STAT4 phosphorylation leading to IFN-γ synthesis (Lertmemongkolchai et al., 2001; Capobianchi et al., 2015). IL-18 stimulates the full activity of NK cells to secrete IFN-γ in response to IL-12 stimulation (Muhlethaler-Mottet et al., 1998; Lykens et al., 2010).

Although type III and type I IFNs share many similarities in their promoter regions and binding sites for transcription factors (Onoguchi et al., 2007; Segundo et al., 2011), for instance, IFN-β and IFN-λ1/2/3 promoters contain IRF- and NF-kβ-binding sites; IFN-α promoters have IRF-binding sites (Iversen and Paludan, 2010; Durbin et al., 2013; Reid and Charleston, 2014), the expression of type III and type I IFNs is regulated through different mechanisms (Numasaki, 2009). For example, type III interferons are produced mainly by antigen-presenting cells and epithelial cells (Chasset and Arnaud, 2017), while the major cell type responsible for type I IFN production is the pDC (Mackern-Oberti et al., 2015; Chasset and Arnaud, 2017). Type I IFNs are induced by mitochondrial-associated MAVS, whereas type III IFNs are stimulated by peroxisome-associated MAVS (Odendall et al., 2014). In addition, IRF1 plays a unique role in type III IFN induction while IRF3 and IRF7 play vital roles in type I IFN production (Österlund et al., 2007).

## Picornavirus Non-Structural Proteins Inhibit IFN Production to Counteract Host Antiviral Responses

While the host secretes IFNs to defense against viral infection, viruses have also developed effective immune evasion mechanisms to counteract the host’s antiviral responses. Numerous studies have demonstrated that picornavirus proteases can cleave adaptors, receptors and regulators involved in the signaling pathways controlling IFN induction to inhibit production of type I IFNs (Wang et al., 2012; Lei et al., 2013). Besides, picornavirus non-structural proteins play important roles in the suppression of IFNs by down-regulating host gene expression and blocking the secretory pathway (Table 1). However, in some picornaviruses, cooperation between non-structural proteins leads to inhibition of IFN induction (Sim et al., 2005; Chase and Semler, 2012).

**Table 1:** Main signaling pathways in suppressing the production of IFNs of non-structural proteins in some picornaviruses.

Non-structural Proteases	Virus	Involved signaling pathways/structure	Type of IFN	Reference
Lpro	FMDV	Cleaving eIF4G, shutting off host cap-dependent mRNA translation, limiting the synthesis of host proteins	Type I IFNs	Devaney et al., 1988; Belsham et al., 2000; de Los Santos et al., 2006
		Degrading NF-κB subunit p65/RelA, ubiquinating RIG-I, TBK1, and TRAF3/6, decreasing IRF-3/7, inhibiting NF-κB	IFN-β	de Los Santos et al., 2007; Wang et al., 2011
		Via Lpro’s catalytic activity and SAP domain	IFN-λ1	Wang et al., 2011
		Disrupting NF-κB and IRF via RIG-I/MDA5	IFN-λ1	Shi et al., 2011; Wang et al., 2011
	EMCV	Lpro hinge domain interacting with Ran and disrupting the Ran GDP-GTP gradient, inhibiting nucleocytoplasmic transport	Unclear	Porter et al., 2006; Ma, 2007; Bacotdavis and Palmenberg, 2013
		Interfering IRF3	IFN-α/β	Hato et al., 2010
	TMEV	Inhibiting IRF-3 dimerization	IFN-β	Stavrou et al., 2010
2A	EV71	Cleaving MAVS and MDA5, preventing IRF3 phosphorylation	Type I IFNs	Feng et al., 2014a
		Inhibiting induction of downstream IFN-stimulated genes, the detailed mechanism is controversial	Unclear	Lu et al., 2012; Liu et al., 2014
		Downregulating KPNA1, reducing formation of the STAT/karyopherin-α1 (KPNA1) complex	Unclear	Wang et al., 2017
		Reducing serine phosphorylation of STAT1 and inactivating extracellular signal-regulated kinases	IFN-γ	Morrison and Racaniello, 2009
	RV	Cleaving MAVS	Unclear	Mukherjee et al., 2011
	CVB3/PV	Cleaving MAVS and MDA5	Type I IFNs	Feng et al., 2014a
2B	HAV	Influencing MAVS function	IFN-β	Ashutosh et al., 2015
		Interfering TBK1/IKK𝜀 kinase complex, inhibiting RIG-I/MDA-5 and IRF3	IFN-β	Paulmann et al., 2008
2C	EV71	Inhibiting IKKβ phosphorylation and NF-κB activation via PP1 binding NF-κB	Unclear	Zheng et al., 2011; Li Q. et al., 2016
		Suppressing p65/p50 dimerization by competing p65 IPT domain, suppressing the activation of NF-κB	Unclear	Du et al., 2015
	CVA16/CVB3	Inhibiting IKKβ phosphorylation and NF-κB activation via PP1 binding	Unclear	Paulmann et al., 2008; Du et al., 2015
3A	FMDV	Reducing expression of MDA5, RIG-I and VISA by decreasing their mRNA levels, inhibiting RLR pathway	IFN-β	Li D. et al., 2016
3C	EV71	Cleaving TRIF and TBK1, inhibiting TLR3 and RIG-I, preventing activation of IRF3 and IRF7	IFN-β	Lei et al., 2010
		Inhibiting IRF7 and IRF9	Type I IFNs	Hung et al., 2011; Lei et al., 2013
		Cleaving TAK1/TAB1/TAB2/TAB3 complex, NF-κB	Unclear	Lei et al., 2014
		Binding with RIG-I, impairing RIG-I’s interaction with MAVS	Type I IFNs	Xu et al., 2014
	CV-A16, CV-A6, EV-D68	Cleaving TAK1 to inhibit the NF-κB response	Unclear	Rui et al., 2017
		Binding with MDA5, inhibiting the interaction with MAVS	Type I IFNs	Rui et al., 2017
	CVB3	Cleaving MAVS and TRIF	Type I IFNs	Mukherjee et al., 2011
	EMCV	Cleaving TANK, disrupting the formation of the TANK–TBK1–IKK𝜀–IRF3 tetramer, decreasing TBK1- and IKK𝜀-mediated IRF3 phosphorylation, impairing the ability of TANK to inhibit TRAF6-mediated NF-κB signaling	Type I IFNs	Huang et al., 2015, 2017
		Blocking formation of SG	Unclear	
		Cleaving IRF3-5D, inhibits JAK-STAT signaling	Type I IFNs	
		Suppressing STAT1 or IRF3 binding to the IFN-β promoter	Type I IFNs	
	FMDV	Cleaving NEMO	Unclear	Zhao et al., 2007
		Cleaving TANK, generating a 15-kDa N-terminal fragment and impairing TANK’s ability to suppress TRAF6-mediated NF-κB signaling	Unclear	Fan et al., 2017
		Suppressing IRF3 by degrading autophagy-related protein ATG5-ATG12	Unclear	Fan et al., 2017
		Degrading KPNA1, blocking STAT1/STAT2 nuclear translocation	Unclear	Du et al., 2014
	SVV	Cleaving MAVS, TRIF, and TANK	Type I IFNs	Qian et al., 2017
		Reducing the expression of IRF3 and IRF7 and phosphorylating them	IFN-α1, IFN-α4, and IFN-β	Xue et al., 2018
	HAV	Cleaving MAVS	Type I IFNs	Yang et al., 2007
		Cleaving NEMO	Unclear	Wang et al., 2014; Xu et al., 2014
		Inhibiting NF-κB activation through cleavage of the TAK1/TAB1/TAB2/TAB3 complex	Unclear	Lei et al., 2013, 2014
3ABC	HAV	Cleaving MAVS and disrupts activation of IRF3 through the RLR pathway	Unclear	Yang et al., 2007; Debing et al., 2014
3CD		Disrupting RIGI/MDA5, inhibiting dimerization of IRF-3 and translocation of IRF-3 to the nucleus	IFN-β	Qu et al., 2011
3D	EV71	Attenuating STAT1 tyrosine phosphorylation	IFN-γ	Wang et al., 2015


### Lpro

#### Lpro of FMDV

Foot-and-mouth disease virus Lpro is a kind of papain-like cysteine protease, which was first identified by Strebel and Beck (1986). FMDV’s RNA genome encodes a polyprotein, Lpro is located near its N-terminus (residue ∼2330) and there are two forms of FMDV Lpro, Laboratory (201 aa) and Lb (173 aa) (Kirchweger et al., 1994). FMDV infection selectively induces IFN-α1 mRNA, and IFN-β mRNA levels become elevated only after a significant duration of infection (24 h) (de Los Santos et al., 2006). By inhibiting IFN production (including type I and type III IFNs) at the transcriptional and translational levels, FMDV Lpro down regulates the host innate immune response to FMDV infection. FMDV Lpro can repress IFN-β transcription by reducing IFN-stimulated gene products (Li D. et al., 2016) decreasing IFN-β mRNA levels during early infection and inhibiting activation of NF-κB via degradation of the NF-κB subunit p65/RelA and ubiquitination of RIG-I, TBK1, and TRAF3/6, resulting decreased IRF-3/7 protein expression (de Los Santos et al., 2007; Wang et al., 2011). Additionally, Lpro contributes to induce the cleavage of host eukaryotic translation initiation factor 4γ (eIF4G) (Devaney et al., 1988; Belsham et al., 2000), shutting off host cap-dependent mRNA translation, thus limiting the synthesis of host proteins (de Los Santos et al., 2006), which may possibly include type I IFNs.

Foot-and-mouth disease virus Lpro also antagonizes IFN-λ1: Lpro’s catalytic activity and SAP domain are involved in the suppression of IFN-λ1 induction (Wang et al., 2011). In addition, by disrupting activation of NF-κB and IRFs and inhibiting IFN-λ1 expression induced by RIG-I/MDA5, FMDV Lpro inhibits IFN-λ1 promoter activation (Wang et al., 2011).

#### Lpro of Cardiovirus

Cardiovirus polyproteins begin with short N-terminal Leader (L) sequences, EMCV Lpro (∼67 residues) and TMEV Lpro (∼76 residues) contains common zinc-finger and acidic domains. Although cardiovirus Lpro is different from FMDV Lpro and does not function as a protease, it represses IFN-α/β synthesis during viral infection. In eukaryotes, nucleocytoplasmic transport of RNA and protein relies on the Ran-GTPase system. EMCV Lpro directly interacts with Ran and disrupts the RanGDP-GTP gradient leading to inhibition of nucleocytoplasmic transport (Porter et al., 2006; Ma, 2007), suppressing the production of IFN. EMCV Lpro hinge domain plays a major role in the interaction with Ran GTPase (Bacotdavis and Palmenberg, 2013). EMCV Lpro interferes with the transactivation function of IRF3 suppressing IRF3-mediated IFN-α/β production (Hato et al., 2010). Studies have demonstrated that TMEV Lpro can block the production of type I IFN at the transcriptional level (Van et al., 2001), this transcriptional inhibition is correlated with inhibition of IRF-3 dimerization (Ricour et al., 2009).

### 2A

#### 2A of Enteroviruses

Enteroviruses 2A has protease activity (Racaniello, 2007), which can not only process the viral polyprotein (Toyoda et al., 1986), but also cleave a variety of host proteins and inhibit the host translation whose function is quite similar to Lpro in FMDV.

*Enterovirus* 71 2A cleaves MAVS from the outer membrane of mitochondria. The cleaved fragments are released into the cytoplasm where they effectively inactivate downstream signaling and cleave MDA5, thus preventing IRF3 phosphorylation, down regulating production of type I IFNs and increasing viral replication (as it was shown in Figure 2; Wang et al., 2013). During this process, EV71 2A cleaves at MAVS residues Gly209, Gly251, and Gly265, with a strong preference for cleavage at Gly251 (Wang et al., 2013). Similarly, Rhinovirus 2A inhibits the production of IFN by cleaving MAVS (Mukherjee et al., 2011). CVB3 (coxsackievirus B3) 2A and poliovirus (PV) also mediate the cleavage of MAVS and MDA5, exerting the same functions in inhibiting type I IFNs (Feng et al., 2014a).

*Enterovirus* 71 can still inhibit induction of downstream IFN-stimulated genes, although the mechanisms of EV71 disruption of IFN signaling have been controversial. Lu et al. (2012) reported that EV71 2A acted as an IFN antagonist and that its protease activity was required for reduction of IFNAR1 levels. By reducing IFNAR1, EV71 inhibits IFN-mediated phosphorylation of Tyk2, Jak1, STAT1, and STAT2. However, Liu et al. (2014) reported that EV71 infection down regulated expression of JAK1 but did not modify expression of IFNAR1. KPNA1 is a nuclear localization signal receptor for p-STAT1. A recent study reported that EV71 infection down regulated expression of KPNA1, reducing the formation of STAT/karyopherin-α1 (KPNA1) complex and resulting reduction of the IFN (Wang et al., 2017).

*Enterovirus* 71 2A attenuates IFN-γ signaling using a mechanism that is different from type I IFNs. 2A can suppress IFN-γ signaling by reducing serine phosphorylation of STAT1 and inactivating extracellular signal-regulated kinases without affecting STAT1 expression (Wang et al., 2015). EV71 2A alters signal transduction of type II IFNs without affecting the protein expression of IFN-γ.

Other genera picornaviruses, such as EMCV, do not encode 2A proteinase. They are sensitive to IFN and are unable to replicate in IFN-pretreated cells. Their 2A might not have function on evading the host immune response (Morrison and Racaniello, 2009).

### 2B

#### 2B of HAV

Hepatitis A virus 2B is a peripheral membrane protein, its coding region has variants (Emerson et al., 1991, 1992, 1993), which makes it significantly larger than 2B in other picornaviruses. HAV 2B was found in close vicinity to the tubular interconnected network of mitochondrial membranes through its ability induce membrane rearrangements resulting in the influence of the production of IFNs (Gosert et al., 2000). HAV 2B suppresses MAVS signaling more effectively with the cooperation of HAV 3ABC (Ashutosh et al., 2015). HAV 2B appears to influence MAVS function without directly affecting the antigenic structure of MAVS (Paulmann et al., 2008); it also interferes with the TBK1/IKK𝜀 kinase complex. Consequently, RIG-I/MDA-5-mediated activation is inhibited and inhibition of IRF-3 signaling results in efficient suppression of IFN-β synthesis (Paulmann et al., 2008).

### 2C

#### 2C of Enteroviruses

Enteroviruses 2C (329 aa and 37.5 kDa), such as EV71 and CVA16 2C ATPase, is not only an RNA helicase but also an ATP-independent RNA chaperone, which is critical for RNA replication and viability of enteroviruses (Xia et al., 2015; Guan et al., 2017). EV71 2C is localized both to the cytoplasm and the nucleus. EV71 2C interacts with protein phosphatase 1 (PP1) catalytic subunit through PP1-docking motifs (residues 1 to 47) located near the N-terminus of EV71 2C. Interactions with IKKβ are formed through a motif (residues 105 to 121) located within N-terminal region of EV71 2C, resulting in formation of a complex between PP1 and IKKβ (Li Q. et al., 2016). PP1 binding is crucial for EV71 2C-mediated inhibition of IKKβ phosphorylation. EV71 2C-mediated PP1 recruitment inhibits IKKβ phosphorylation, NF-κB activation and NF-κB signaling pathway-induced IFN production (Zheng et al., 2011; Li Q. et al., 2016). Other enteroviruses, such as PV, coxsackie A virus 16 (CVA16), and coxsackie B virus 3 (CVB3) also exploit this mechanism to inhibit the production of IFN (Li Q. et al., 2016).

Additionally, EV71 2C (residues 105–125 and 126–263) is capable to suppress p65/p50 dimerization by competing p65 IPT domain in association with p50, suppressing the activation of NF-κB and IFN (Du et al., 2015).

### 3A

#### 3A of FMDV

Foot-and-mouth disease virus 3A is a partially conserved protein, it has no homologous sequence to any other known proteases, which is unique among the picornaviruses. A recent study revealed that FMDV 3A down regulates FMDV-associated IFN-β induction via FMDV 3A inhibition of RLR-mediated IFN-β induction (Li D. et al., 2016). Residues 103–153 near 3A’s N-terminus interact with MDA5, RIG-I and VISA, and a 102-residue region near the N-terminus mediates inhibition of the IFN-β signaling pathway (Li D. et al., 2016). FMDV 3A reduces expression of MDA5, RIG-I and VISA by decreasing their mRNA levels (Li D. et al., 2016). This finding not only reveals a novel mechanism of FMDV 3A-mediated evasion of host innate immunity but also provide a new thought to explore this kind of non-structural proteins in other picornaviruses.

### 3C

Picornavirus 3C is a unique cysteine protease that combines features of both serine and cysteine proteases (Di et al., 2016). Although 3C has similar spatial structures among all picornaviruses, and can inhibit IFN expression through similar pathways, including the NF-κB, Jak/STAT and IRF pathways, its specific sites of action are different.

#### 3C of Enteroviruses

*Enterovirus* 71 3C is one of the most common functional proteins, which has been most widely studied in enteroviruses. EV71 3C inhibits induction of IFN by RIG-I or TLR3 and prevents activation of IRF3 and IRF7. Upon viral infection, TLR3 recruits TRIF (TIR domain-containing adaptor inducing IFN-β) and TBK1, which phosphorylate IRF3 and IRF7 (Lei et al., 2010). The TRIF Q312–S313 junction is critical for its cleavage by EV71 3C. EV71 3C-induced TRIF cleavage blocks IFN-β and NF-κB activation by TRIF (Lei et al., 2011). EV 71 3C can directly inhibit IRF7 and IRF9, repressing type I IFN production (Hung et al., 2011; Lei et al., 2013). EV71 3C protease activity is necessary to cleave IRF7. EV71 3C cleaves IRF7 at the Q189–S190 junction, yielding two fragments that are unable to stimulate IFN production (Lei et al., 2013).

Likewise, EV71 3C reduces IFN production by inhibiting activation of NF-κB (Lei et al., 2014). Transforming growth factor-β-activated kinase 1 (TAK1), TAK1-binding protein (TAB)1, TAB2, and TAB3 are all required for activation of downstream NF-κB. In mammalian cells, TAK1 binds to TAB1, forming TAK1-TAB1 complex. Thereafter, TAB2 and TAB3 are recruited to TAK1-TAB1 complex forming TAK1/TAB1/TAB2/TAB3 complex. This complex activates p38, IKKαβ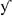 and c-Jun N-terminal kinase (JNK), thus inducing IFN production (Lei et al., 2014). EV71 3C cleaves TAK1 at the Q360–S361 junction yielding smaller products of about 30 kDa. The TAB1 Q414–G415 and Q451–S452 junctions are EV71 3C cleavage sites; cleavage results in about 45 kDa and 50 kDa products. EV71 3C cleaves TAB2 at the Q113–S114 junction. EV71 3C cleaves TAB3 at the Q173–G174 and Q343–G344 junctions, resulting in about 45 kDa and 60 kDa products. Cleavage disrupts the TAK1/TAB1/TAB2/TAB3 complex and reduces IFN production. It should be noted that TAB2 has NF-κB-activating function, but cleavage by EV71 3C impairs this activity (Lei et al., 2014). On the other hand, CVA-16, CV-A6, and EV-D68 3C cleave TAK1 to inhibit the NF-κB response (Rui et al., 2017).

Upon viral infection, EV71 3C can directly bind to RIG-I, impairing RIG-I’s interaction with MAVS and inhibiting RIG-I-mediated type I IFN responses. It has been reported that ubiquitination of RIG-I is controlled by a tumor suppressor called CYLD (Xu et al., 2014). CYLD is a target of miR-526a, a potent IFN-β inducer, and miR-526a upregulation during viral infection is partially mediated by IRF7. By suppressing CYLD expression, miR-526a positively regulates VSV-associated type I IFN production. EV71 3C inhibits production of type I IFN by blocking miR-526a upregulation and CYLD downregulation.

CV-A16, CV-A6, and EV-D68 3C can bind to MDA5 and inhibit the interaction with MAVS, thus blocking the production of type I IFN (Rui et al., 2017). CVB3 3C also cleaves MAVS and Toll/IL-1 receptor domain-containing adaptor inducing interferon-beta (TRIF) at specific sites and inhibits the induction of type I IFN (Mukherjee et al., 2011).

#### 3C of EMCV

*Encephalomyocarditis virus* 3C is the only cysteine protease encoded by the viral genome, and it has a high degree of substrate specificity, besides Lpro, EMCV 3C is another antagonist. TANK is an NF-κB activator, TRAF6 serves as a platform to recruit the IKK complex and kinase TAK1, and TANK negatively regulates this function (Papon et al., 2009). EMCV 3C can cleave TANK at Gln291 and Gln197 (Huang et al., 2015), disrupting formation of the TANK–TBK1–IKK𝜀–IRF3 tetramer, decreasing TBK1- and IKK𝜀-mediated IRF3 phosphorylation, impairing the ability of TANK to inhibit TRAF6-mediated NF-κB signaling, and reducing type I IFN production (Huang et al., 2015, 2017). SG is the location for efficient interaction between viral RNA and RLRs; EMCV 3C can also block formation of SG to inhibit activation of IFN genes (Huang et al., 2017). By cleaving IRF3-5D and other key proteins, EMCV 3C inhibits JAK-STAT signaling, suppressing type I IFN production (Huang et al., 2017). EMCV 3C may also suppress STAT1 or IRF3 binding to the IFN-β promoter to inhibit type I IFN production (Huang et al., 2017).

#### 3C of FMDV

Foot-and-mouth disease virus 3C plays important roles in disrupting the translational system of the host and can negatively regulate innate immune signaling by degrading essential molecules in different pathways (Ma et al., 2018a). FMDV 3C has the ability to cleave NEMO at Gln383 (Zhao et al., 2007); cleavage impairs NEMO-mediated IFN production and its ability to act as a signaling adaptor in the RIG-I/MDA5 pathway (Wang et al., 2012). Moreover, FMDV 3C cleaves TANK, generating a 15-kDa N-terminal fragment and impairing TANK’s ability to suppress TRAF6-mediated NF-κB signaling (Fan et al., 2017).

Under normal conditions, ATG5-ATG12 promotes activation of IRF3 and phosphorylation of TBK1 by preventing TRAF3 degradation, resulting in enhanced expression of IFN-β (Fan et al., 2017). FMDV suppresses IRF3 by degradation of autophagy-related protein ATG5-ATG12 to attenuate production of IFN via 3C (Fan et al., 2017).

Karyopherin α1 (KPNA1) is the nuclear localization signal receptor for STAT1. FMDV 3C interferes with the JAK-STAT signaling pathway by degrading KPNA1, blocking STAT1/STAT2 nuclear translocation and inhibiting IFN signaling (Du et al., 2014).

#### 3C of SVV

Seneca Valley virus (SVV) is most closely related to *Cardiovirus* (Hales et al., 2008). SVV 3C has a conserved catalytic box with His and Cys residues (Qian et al., 2016), which is similar to FMDV Lpro. SVV 3C can inhibit the production of type I IFN by directly cleaving MAVS, TRIF, and TANK (Qian et al., 2016). In addition, a recent result indicates that SVV 3C reduces the expression of IRF3 and IRF7 and phosphorylates them and then blocks the transcription of IFN-β, IFN-α1, IFN-α4, and ISG54 (Xue et al., 2018).

#### 3C of HAV

Hepatitis A virus 3C is a cysteine proteinase which is responsible for most cleavages within the viral polyprotein (Schultheiss et al., 1994, 1995). HAV 3C cleaves MAVS at Gln428 to inhibit type I IFN production (Yang et al., 2007). Similar to FMDV 3C, HAV 3C also cleaves NEMO, impairing NEMO-mediated IFN production and its ability to act as a signaling adaptor in the RIG-I/MDA5 pathway (Wang et al., 2014; Xu et al., 2014). Moreover, HAV 3C inhibits NF-κB activation through cleavage of the TAK1/TAB1/TAB2/TAB3 complex, inhibiting the induction of IFNs (Lei et al., 2013, 2014).

#### 3ABC and 3CD of HAV

Processing intermediate HAV 3ABC and 3CD are both unique and have proteolytically activities in particle assembly (Probst et al., 1998). HAV 3ABC is a precursor cysteine protease. 3ABC cleaves MAVS and disrupts activation of IRF3 through the RLR pathway in mitochondria (Yang et al., 2007; Debing et al., 2014). With the help of the transmembrane domain of 3A, 3ABC localizes to mitochondria. MAVS cleavage also requires the protease activity of 3C (Yang et al., 2007). This feature of 3ABC is unique among picornaviruses.

Hepatitis A virus 3CD is the processing intermediate of 3ABCD. HAV 3CD disrupts RIGI/MDA5, inhibits dimerization of IRF-3 and translocation of IRF-3 to the nucleus, and impairs IFN-β promoter activation (Qu et al., 2011).

### 3D

#### 3D of EV71

*Enterovirus* 71 3D is a kind of RNA-dependent RNA polymerase (Jiang et al., 2011; Sun et al., 2012). Wang et al. (2015) found that without interfering with IFN-γ receptor expression, EV71 3D can attenuate STAT1 tyrosine phosphorylation resulting in defective IFN-γ signaling. The detailed signaling pathway how 3D regulate STAT1 need further investigation, Wang et al. (2015) guess that the function of EV71 3D may similar to EV71 2A, as a viral factor for immune-editing.

## Outlook

The interactions between picornaviruses and host defenses are complex and diverse. Moreover, viruses have developed multiple strategies to evade the host’s innate immune system. To date, some of these strategies have been uncovered and significant progress has been achieved in understanding signaling pathways related to immune evasion. For example, the mechanism underlying inhibition by some non-structural proteins of IFN production in picornaviruses have been well studied. However, what we know today just represent a drop in the bucket, and we still need to understand the viral strategies involved in antagonizing the host’s innate immune system. For example, SVV 3C has similar conserved catalytic box and similar function to FMDV Lpro in antagonizing the innate immune response and whether SVV 3C has other similar function to FMDV Lpro need further research. In addition, there are many similarities between different genera of picornaviruses. However, further efforts should be made to explore key mechanisms underlying inhibition by some non-structural proteins of IFN production, such as 2B, 2C, 3A, and 3D, across all picornavirus.

Recently, it has been discovered that some picornaviruses only cause an acute and self-limiting infection without major pathogenesis in hosts requiring more research on therapeutic approach (Weinberg and Morris, 2016; Ma et al., 2018b). The role of non-structural proteins in such picornaviruses may make contributions to better understand not only the therapeutic antiviral activity of IFNs, but also may reveal how these proteins (with or without protease activities) influence and control the IFN signaling transduction *in vivo*.

## Author Contributions

YW is the first author of this article and wrote the manuscript. LM, LS, and SS had made contributions to this article. XL and YL had revised this article.

## Conflict of Interest Statement

The authors declare that the research was conducted in the absence of any commercial or financial relationships that could be construed as a potential conflict of interest.

## References

[B1] Akira S. Uematsu S. Takeuchi O. (2006). Pathogen recognition and innate immunity. *Cell* 124 783–801. 10.1016/j.cell.2006.02.015 16497588

[B2] Ank N. West H. Bartholdy C. Eriksson K. Thomsen A. R. Paludan S. R. (2006). Lambda interferon (IFN-lambda), a type III IFN, is induced by viruses and IFNs and displays potent antiviral activity against select virus infections in vivo. *J. Virol.* 80 4501–4509. 10.1128/JVI.80.9.4501-4509.2006 16611910 PMC1472004

[B3] Argos P. Kamer G. Nicklin M. J. Wimmer E. (1984). Similarity in gene organization and homology between proteins of animal picornaviruses and a plant comovirus suggest common ancestry of these virus families. *Nucleic Acids Res.* 12 7251–7267. 10.1093/nar/12.18.7251 6384934 PMC320155

[B4] Ashutosh S. Debajit D. Kamalika B. Anshu N. Manidipa B. (2015). The C-terminal region of the non-structural protein 2B from Hepatitis A Virus demonstrates lipid-specific viroporin-like activity. *Sci. Rep.* 5 :15884. 10.1038/srep15884 26515753 PMC4626808

[B5] Bacotdavis V. R. Palmenberg A. C. (2013). Encephalomyocarditis virus leader protein hinge domain is responsible for interactions with ran GTPase. *Virology* 443 177–185. 10.1016/j.virol.2013.05.002 23711384 PMC3724357

[B6] Barbé F. Douglas T. Saleh M. (2014). Advances in Nod-like receptors (NLR) biology. *Cytokine Growth Factor Rev.* 25 681–697. 10.1016/j.cytogfr.2014.07.001 25070125

[B7] Belsham G. J. McInerney G. M. Ross-Smith N. (2000). Foot-and-mouth disease virus 3C protease induces cleavage of translation initiation factors eIF4A and eIF4G within infected cells. *J. Virol.* 74 272–280. 10.1128/JVI.74.1.272-280.2000 10590115 PMC111537

[B8] Blaszczyk K. Nowicka H. Kostyrko K. Antonczyk A. Wesoly J. Bluyssen H. A. (2016). The unique role of STAT2 in constitutive and IFN-induced transcription and antiviral responses. *Cytokine Growth Factor Rev.* 29 71–81. 10.1016/j.cytogfr.2016.02.010 27053489

[B9] Buenz E. J. Howe C. L. (2006). Picornaviruses and cell death. *Trends Microbiol.* 14 28–36. 10.1016/j.tim.2005.11.003 16337385

[B10] Capobianchi M. R. Uleri E. Caglioti C. Dolei A. (2015). Type I IFN family members: similarity, differences and interaction. *Cytokine Growth Factor Rev.* 26 103–111. 10.1016/j.cytogfr.2014.10.011 25466633 PMC7108279

[B11] Chase A. J. Semler B. L. (2012). Viral subversion of host functions for picornavirus translation and RNA replication. *Future Virol.* 7 179–191. 10.2217/fvl.12.2 23293659 PMC3535308

[B12] Chasset F. Arnaud L. (2017). Targeting interferons and their pathways in systemic lupus erythematosus. *Autoimmun. Rev.* 17 44–52. 10.1016/j.autrev.2017.11.009 29108825

[B13] Coccia E. M. Battistini A. (2015). Early IFN type I response: learning from microbial evasion strategies. *Semin. Immunol.* 27 85–101. 10.1016/j.smim.2015.03.005 25869307 PMC7129383

[B14] de Los Santos T. de Avila Botton S. Weiblen R. Grubman M. J. (2006). The leader proteinase of foot-and-mouth disease virus inhibits the induction of beta interferon mRNA and blocks the host innate immune response. *J. Virol.* 80 1906–1914. 10.1128/JVI.80.4.1906-1914.2006 16439546 PMC1367153

[B15] de Los Santos T. Diazsan S. F. Grubman M. J. (2007). Degradation of nuclear factor kappa B during foot-and-mouth disease virus infection. *J. Virol.* 81 12803–12815. 10.1128/JVI.01467-07 17881445 PMC2169123

[B16] Debing Y. Neyts J. Thibaut H. J. (2014). Molecular biology and inhibitors of hepatitis A virus. *Med. Res. Rev.* 34 895–917. 10.1002/med.21292 23722879 PMC7168461

[B17] Devaney M. A. Vakharia V. N. Lloyd R. E. Ehrenfeld E. Grubman M. J. (1988). Leader protein of foot-and-mouth disease virus is required for cleavage of the p220 component of the cap-binding protein complex. *J. Virol.* 1988 4407–4409. 2845152 10.1128/jvi.62.11.4407-4409.1988 PMC253884

[B18] Di S. Chen S. Cheng A. Wang M. (2016). Roles of the picornaviral 3C proteinase in the viral life cycle and host cells. *Viruses* 8 :82. 10.3390/v8030082 26999188 PMC4810272

[B19] Du H. Yin P. Yang X. Zhang L. Jin Q. Zhu G. (2015). Enterovirus 71 2C protein inhibits NF-κB activation by binding to RelA(p65). *Sci. Rep.* 5 :14302. 10.1038/srep14302 26394554 PMC4585786

[B20] Du Y. Bi J. Liu J. Liu X. Wu X. Jiang P. (2014). 3Cpro of foot-and-mouth disease virus antagonizes the interferon signaling pathway by blocking STAT1/STAT2 nuclear translocation. *J. Virol.* 88 4908–4920. 10.1128/JVI.03668-13 24554650 PMC3993825

[B21] Durbin R. K. Kotenko S. V. Durbin J. E. (2013). Interferon induction and function at the mucosal surface. *Immunol. Rev.* 255 25–39. 10.1111/imr.12101 23947345 PMC5972370

[B22] Ehrenfeld E. Domingo E. Roos R. P. Ehrenfeld E. Domingo E. Roos R. P. (2010). *The Picornaviruses.* Washington, DC: ASM Press 10.1128/9781555816537

[B23] Emerson S. U. Huang Y. K. McRill C. Lewis M. Purcell R. H. (1992). Mutations in both the 2B and 2C genes of hepatitis A virus are involved in adaptation to growth in cell culture. *J. Virol.* 66 650–654. 1309907 10.1128/jvi.66.2.650-654.1992 PMC240763

[B24] Emerson S. U. Huang Y. K. Purcell R. H. (1993). 2B and 2C mutations are essential but mutations throughout the genome of HAV contribute to adaptation to cell culture. *Virology* 194 475–480. 10.1006/viro.1993.1286 8389072

[B25] Emerson S. U. McRill C. Rosenblum B. Feinstone S. M. Purcell R. H. (1991). Mutations responsible for adaptation of hepatitis A virus to efficient growth in cell culture. *J. Virol.* 65 4882–4886. 1651411 10.1128/jvi.65.9.4882-4886.1991 PMC248948

[B26] Fan X. Han S. Yan D. Gao Y. Wei Y. Liu X. (2017). Foot-and-mouth disease virus infection suppresses autophagy and NF-ˆeB antiviral responses via degradation of ATG5-ATG12 by 3Cpro. *Cell Death Dis.* 8 :e2561. 10.1038/cddis.2016.489 28102839 PMC5386389

[B27] Feng Q. Langereis M. A. Lork M. Mai N. Hato S. V. Lanke K. (2014a). Enterovirus 2Apro targets MDA5 and MAVS in infected cells. *J. Virol.* 88 3369–3378. 10.1128/JVI.02712-13 24390337 PMC3957915

[B28] Feng Q. Langereis M. A. van Kuppeveld F. J. (2014b). Induction and suppression of innate antiviral responses by picornaviruses. *Cytokine Growth Factor Rev.* 25 577–585. 10.1016/j.cytogfr.2014.07.003 25086453 PMC7172595

[B29] Fensterl V. Chattopadhyay S. Sen G. C. (2015). No love lost between viruses and interferons. *Annu. Rev. Virol.* 2 549–572. 10.1146/annurev-virology-100114-055249 26958928 PMC9549753

[B30] Gonzáleznavajas J. M. Lee J. David M. Raz E. (2012). Immunomodulatory functions of type I interferons. *Nat. Rev. Immunol.* 12 125–135. 10.1038/nri3133 22222875 PMC3727154

[B31] Gosert R. Egger D. Bienz K. (2000). A cytopathic and a cell culture adapted hepatitis A virus strain differ in cell killing but not in intracellular membrane rearrangements. *Virology* 266 157–169. 10.1006/viro.1999.0070 10612670

[B32] Guan H. Tian J. Qin B. Wojdyla J. A. Wang B. Zhao Z. (2017). Crystal structure of 2C helicase from Enterovirus 71. *Sci. Adv.* 3 :e1602573. 10.1126/sciadv.1602573 28508043 PMC5409451

[B33] Hales L. M. Knowles N. J. Reddy P. S. Xu L. Hay C. Hallenbeck P. L. (2008). Complete genome sequence analysis of Seneca Valley virus-001, a novel oncolytic picornavirus. *J. Gen. Virol.* 89(Pt 5), 1265–1275. 10.1099/vir.0.83570-0 18420805

[B34] Hato S. V. Ricour C. Schulte B. M. Lanke K. H. De B. M. Zoll J. (2010). The mengovirus leader protein blocks interferon-alpha/beta gene transcription and inhibits activation of interferon regulatory factor 3. *Cell Microbiol.* 9 2921–2930. 10.1111/j.1462-5822.2007.01006.x 17991048

[B35] Hernandez P. P. Mahlakoiv T. Yang I. Schwierzeck V. Nguyen N. Guendel F. (2015). Interferon-lambda and interleukin 22 act synergistically for the induction of interferon-stimulated genes and control of rotavirus infection. *Nat. Immunol.* 16 698–707. 10.1038/ni.3180 26006013 PMC4589158

[B36] Hillyer P. Mane V. P. Schramm L. M. Puig M. Verthelyi D. Chen A. (2012). Expression profiles of human interferon-alpha and interferon-lambda subtypes are ligand- and cell-dependent. *Immunol. Cell Biol.* 90 774–783. 10.1038/icb.2011.109 22249201 PMC3442264

[B37] Honda K. Taniguchi T. (2006). IRFs: master regulators of signalling by Toll-like receptors and cytosolic pattern-recognition receptors. *Nat. Rev. Immunol.* 6 644–658. 10.1038/nri1900 16932750

[B38] Huang L. Liu Q. Zhang L. Zhang Q. Hu L. Li C. (2015). Encephalomyocarditis virus 3C protease relieves TRAF family member-associated NF-κB activator (TANK) inhibitory effect on TRAF6-mediated NF-κB signaling through cleavage of TANK. *J. Biol. Chem.* 290 27618–27632. 10.1074/jbc.M115.660761 26363073 PMC4646013

[B39] Huang L. Xiong T. Yu H. Zhang Q. Zhang K. Li C. (2017). Encephalomyocarditis virus 3C protease attenuates type I interferon production through disrupting TANK-TBK1-IKK𝜀-IRF3 complex. *Biochem. J.* 474 2051–2065. 10.1042/BCJ20161037 28487378 PMC5465970

[B40] Hung H. C. Wang H. C. Shih S. R. Teng I. Tseng C. P. Hsu J. T. A. (2011). Synergistic inhibition of Enterovirus 71 replication by interferon and rupintrivir. *J. Infect. Dis.* 203 1784–1790. 10.1093/infdis/jir174 21536800

[B41] Isaacs A. Lindenmann J. (2015). Pillars article: virus interference. I. The interferon. Proc R Soc Lond B Biol Sci. 1957. 147: 258-267. *J. Immunol.* 195 1911–1920. 26297790

[B42] Iversen M. B. Paludan S. R. (2010). Mechanisms of type III interferon expression. *J. Interferon Cytokine Res.* 30 573–578. 10.1089/jir.2010.0063 20645874

[B43] Jiang H. Weng L. Zhang N. Arita M. Li R. Chen L. (2011). Biochemical characterization of Enterovirus 71 3D RNA polymerase. *Biochim. Biophys. Acta* 1809 211–219. 10.1016/j.bbagrm.2011.01.001 21220056

[B44] Kindler E. Thiel V. Weber F. (2016). Interaction of SARS and MERS Coronaviruses with the antiviral interferon response. *Adv. Virus Res.* 96 219–243. 10.1016/bs.aivir.2016.08.006 27712625 PMC7112302

[B45] Kirchweger R. Ziegler E. Lamphear B. J. Waters D. Liebig H. D. Sommergruber W. (1994). Foot-and-mouth disease virus leader proteinase: purification of the Lb form and determination of its cleavage site on eIF-4 gamma. *J. Virol.* 68 5677–5684. 8057448 10.1128/jvi.68.9.5677-5684.1994 PMC236969

[B46] Klotz D. Baumgãrtner W. Gerhauser I. (2017). Type I interferons in the pathogenesis and treatment of canine diseases. *Vet. Immunol. Immunopathol.* 191 80–93. 10.1016/j.vetimm.2017.08.006 28895871

[B47] Lei X. Han N. Xiao X. Jin Q. He B. Wang J. (2014). Enterovirus 71 3C inhibits cytokine expression through cleavage of the TAK1/TAB1/TAB2/TAB3 complex. *J. Virol.* 88 9830–9841. 10.1128/JVI.01425-14 24942571 PMC4136319

[B48] Lei X. Liu X. Ma Y. Sun Z. Yang Y. Jin Q. (2010). The 3C protein of Enterovirus 71 inhibits retinoid acid-inducible gene I-mediated interferon regulatory factor 3 activation and type I interferon responses. *J. Virol.* 84 8051–8061. 10.1128/JVI.02491-09 20519382 PMC2916543

[B49] Lei X. Sun Z. Liu X. Jin Q. He B. Wang J. (2011). Cleavage of the adaptor protein TRIF by Enterovirus 71 3C inhibits antiviral responses mediated by toll-like receptor 3. *J. Virol.* 85 8811–8818. 10.1128/JVI.00447-11 21697485 PMC3165803

[B50] Lei X. Xiao X. Xue Q. Jin Q. He B. Wang J. (2013). Cleavage of interferon regulatory factor 7 by Enterovirus 71 3C suppresses cellular responses. *J. Virol.* 87 1690–1698. 10.1128/JVI.01855-12 23175366 PMC3554134

[B51] Lertmemongkolchai G. Cai G. Hunter C. A. Bancroft G. J. (2001). Bystander activation of CD8^+^ T cells contributes to the rapid production of IFN-gamma in response to bacterial pathogens. *J. Immunol.* 166 1097–1105. 10.4049/jimmunol.166.2.1097 11145690

[B52] Li D. Lei C. Xu Z. Yang F. Liu H. Zhu Z. (2016). Foot-and-mouth disease virus non-structural protein 3A inhibits the interferon-β signaling pathway. *Sci. Rep.* 6 :21888. 10.1038/srep21888 26883855 PMC4756384

[B53] Li Q. Zheng Z. Liu Y. Zhang Z. Liu Q. Meng J. (2016). 2C proteins of Enteroviruses suppress IKKβ phosphorylation by recruiting protein phosphatase 1. *J. Virol.* 90 5141–5151. 10.1128/JVI.03021-15 26962213 PMC4859720

[B54] Liu Y. Zhang Z. Zhao X. Yu R. Zhang X. Wu S. (2014). Enterovirus 71 inhibits cellular type I interferon signaling by downregulating JAK1 protein expression. *Viral Immunol.* 27 267–276. 10.1089/vim.2013.0127 24905060

[B55] Lu J. Yi L. Zhao J. Yu J. Chen Y. Lin M. C. (2012). Enterovirus 71 disrupts interferon signaling by reducing the level of interferon receptor 1. *J. Virol.* 86 3767–3776. 10.1128/JVI.06687-11 22258259 PMC3302529

[B56] Lykens J. E. Terrell C. E. Zoller E. E. Divanovic S. Trompette A. Karp C. L. (2010). Mice with a selective impairment of IFN-gamma signaling in macrophage lineage cells demonstrate the critical role of IFN-gamma-activated macrophages for the control of protozoan parasitic infections in vivo. *J. Immunol.* 184 877–885. 10.4049/jimmunol.0902346 20018611 PMC2886308

[B57] Ma Q. H. (2007). Small GTP-binding proteins and their functions in plants. *J. Plant Growth Regul.* 26 369–388. 10.1007/s00344-007-9022-7

[B58] Ma X. X. Ma L. N. Chang Q. Y. Ma P. Li L. J. Wang Y. Y. (2018a). Type I interferon induced and antagonized by foot-and-mouth disease virus. *Front. Microbiol.* 13 :1862. 10.3389/fmicb.2018.01862 30150977 PMC6099088

[B59] Ma X. X. Ma Z. R. Pan Q. W. (2018b). The challenges of long-term transcriptional gene silencing by RNA viruses. *Trends Biochem. Sci.* 43 649–650. 10.1016/j.tibs.2018.06.010 30041840

[B60] Mackern-Oberti J. P. Llanos C. Vega F. Salazar-Onfray F. Riedel C. A. Bueno S. M. (2015). Role of dendritic cells in the initiation, progress and modulation of systemic autoimmune diseases. *Autoimmun. Rev.* 14 127–139. 10.1016/j.autrev.2014.10.010 25449681

[B61] Mahlakoiv T. Hernandez P. Gronke K. Diefenbach A. Staeheli P. (2015). Leukocyte derived IFN-alpha/beta and epithelial IFN-lambda constitute a compartmentalized mucosal defense system that restricts enteric virus infections. *PLoS Pathog.* 11 :e1004782. 10.1371/journal.ppat.1004782 25849543 PMC4388470

[B62] Morrison J. M. Racaniello V. R. (2009). Proteinase 2Apro is essential for Enterovirus replication in type I interferon-treated cells. *J. Virol.* 83 4412–4422. 10.1128/JVI.02177-08 19211759 PMC2668472

[B63] Muhlethaler-Mottet A. Di B. W. Otten L. A. Mach B. (1998). Activation of the MHC class II transactivator CIITA by interferon-gamma requires cooperative interaction between Stat1 and USF-1. *Immunity* 8 157–166. 10.1016/S1074-7613(00)80468-9 9491997

[B64] Mukherjee A. Morosky S. A. Delorme-Axford E. Dybdahl-Sissoko N. Oberste M. S. Wang T. (2011). The coxsackievirus B 3C protease cleaves MAVS and TRIF to attenuate host type I interferon and apoptotic signaling. *PLoS Pathog.* 7 :e1001311. 10.1371/journal.ppat.1001311 21436888 PMC3059221

[B65] Nagano Y. Kojima Y. (1954). Immunizing property of vaccinia virus inactivated by ultraviolets rays. *C. R. Seances Soc. Biol. Fil.* 148 1700–1702. 14364998

[B66] Numasaki M. (2009). “IL-28 and IL-29 in regulation of antitumor immune response and induction of tumor regression,” in *Targeted Cancer Immune Therapy*, eds Lustgarten J. Cui Y. Li S. (New York, NY: Springer-Verlag New York), 75–95. 10.1007/978-1-4419-0170-5_5

[B67] Odendall C. Dixit E. Stavru F. Bierne H. Franz K. M. Durbin A. F. (2014). Diverse intracellular pathogens activate type III interferon expression from peroxisomes. *Nat. Immunol.* 15 717–726. 10.1038/ni.2915 24952503 PMC4106986

[B68] Olière S. Douville R. Sze A. Belgnaoui S. M. Hiscott J. (2011). Modulation of innate immune responses during human T-cell leukemia virus (HTLV-1) pathogenesis. *Cytokine Growth Factor Rev.* 22 197–210. 10.1016/j.cytogfr.2011.08.002 21924945

[B69] Onoguchi K. Yoneyama M. Takemura A. Akira S. Taniguchi T. Namiki H. (2007). Viral infections activate types I and III interferon genes through a common mechanism. *J. Biol. Chem.* 282 7576–7581. 10.1074/jbc.M608618200 17204473

[B70] Österlund P. I. Pietilä T. E. Veckman V. Kotenko S. V. Julkunen I. (2007). IFN regulatory factor family members differentially regulate the expression of type III IFN (IFN-λ) genes. *J. Immunol.* 179 3434–3442. 10.4049/jimmunol.179.6.3434 17785777

[B71] Papon L. Oteiza A. Imaizumi T. Kato H. Brocchi E. Lawson T. G. (2009). The viral RNA recognition sensor RIG-I is degraded during Encephalomyocarditis virus (EMCV) infection. *Virology* 393 311–318. 10.1016/j.virol.2009.08.009 19733381

[B72] Paulmann D. Magulski T. Schwarz R. Heitmann L. Flehmig B. Vallbracht A. (2008). Hepatitis A virus protein 2B suppresses beta interferon (IFN) gene transcription by interfering with IFN regulatory factor 3 activation. *J. Gen. Virol.* 89 1593–1604. 10.1099/vir.0.83521-0 18559929

[B73] Peng Q. Lan X. Wang C. Ren Y. Yue N. Wang J. (2017). Kobuvirus VP3 protein restricts the IFN-β-triggered signaling pathway by inhibiting STAT2-IRF9 and STAT2-STAT2 complex formation. *Virology* 507 161–169. 10.1016/j.virol.2017.04.023 28441586

[B74] Pervolaraki K. Stanifer M. L. Munchau S. Renn L. A. Albrecht D. Kurzhals S. (2017). Type I and type III interferons display different dependency on mitogen-activated protein kinases to mount an antiviral state in the human gut. *Front. Immunol.* 8 :459. 10.3389/fimmu.2017.00459 28484457 PMC5399069

[B75] Pham A. M. Maria F. G. S. Lahiri T. Friedman E. Marié I. J. Levy D. E. (2016). PKR transduces MDA5-dependent signals for type I IFN induction. *PLoS Pathog.* 12 :e1005489. 10.1371/journal.ppat.1005489 26939124 PMC4777437

[B76] Platanias L. C. (2005). Mechanisms of type-I- and type-II-interferon-mediated signalling. *Nat. Rev. Immunol.* 5 375–386. 10.1038/nri1604 15864272

[B77] Porter F. W. Bochkov Y. A. Albee A. J. Wiese C. Palmenberg A. C. (2006). A picornavirus protein interacts with Ran-GTPase and disrupts nucleocytoplasmic transport. *Proc. Natl. Acad. Sci. U.S.A.* 103 12417–12422. 10.1073/pnas.0605375103 16888036 PMC1567894

[B78] Probst C. Jecht M. Gauss-Muller V. (1998). Processing of proteinase precursors and their effect on hepatitis A virus particle formation. *J. Virol.* 72 8013–8020. 9733840 10.1128/jvi.72.10.8013-8020.1998 PMC110137

[B79] Qian S. Fan W. Liu T. Wu M. Zhang H. Cui X. (2017). Seneca valley virus suppresses host type I interferon production by targeting adaptor proteins MAVS, TRIF, and TANK for cleavage. *J. Virol.* 91 : e00823-17. 10.1128/JVI.00823-17 28566380 PMC5533933

[B80] Qian S. Fan W. Qian P. Chen H. Li X. (2016). Isolation and full-genome sequencing of Seneca Valley virus in piglets from China, 2016. *Virol. J.* 13 :173. 10.1186/s12985-016-0631-2 27756396 PMC5069920

[B81] Qu L. Feng Z. Yamane D. Liang Y. Lanford R. E. Li K. (2011). Disruption of TLR3 signaling due to cleavage of TRIF by the hepatitis A virus protease-polymerase processing intermediate, 3CD. *PLoS Pathog.* 7 :e1002169. 10.1371/journal.ppat.1002169 21931545 PMC3169542

[B82] Racaniello V. (2007). “Picornaviridae: the viruses and their replication,” in *Fields Virology*, 5th Edn, eds Knipe D. M. Howley P. M. (Philadelphia, PA: Lippincott Williams & Wilkins), 795–838.

[B83] Raki M. Beitnes A. C. Lundin K. E. Jahnsen J. Jahnsen F. L. Sollid L. M. (2013). Plasmacytoid dendritic cells are scarcely represented in the human gut mucosa and are not recruited to the celiac lesion. *Mucosal Immunol.* 6 985–992. 10.1038/mi.2012.136 23340820

[B84] Reid E. Charleston B. (2014). Type I and III interferon production in response to RNA viruses. *J. Interferon Cytokine Res.* 34 649–658. 10.1089/jir.2014.0066 24956361

[B85] Ricour C. Delhaye S. Hato S. V. Olenyik T. D. Michel B. van Kuppeveld F. J. (2009). Inhibition of mRNA export and dimerization of interferon regulatory factor 3 by Theiler’s virus leader protein. *J. Gen. Virol.* 90 177–186. 10.1099/vir.0.005678-0 19088287 PMC2632858

[B86] Rui Y. Su J. Wang H. Chang J. Wang S. Zheng W. (2017). Disruption of MDA5 mediated innate immune responses by the 3C proteins of Coxsackievirus A16, Coxsackievirus A6, and Enterovirus D68. *J. Virol.* 91 : e00546-17. 10.1128/JVI.00546-17 28424289 PMC5469270

[B87] Schroder K. Hertzog P. J. Ravasi T. Hume D. A. (2004). Interferon: an overview of signals, mechanisms and functions. *J. Leukoc. Biol.* 75 163–189. 10.1189/jlb.0603252 14525967

[B88] Schultheiss T. Kusov Y. Y. Gauss-Muller V. (1994). Proteinase 3C of hepatitis A virus (HAV) cleaves the HAV polyprotein P2-P3 at all sites including VP1/2A and 2A/2B. *Virology* 198 275–281. 10.1006/viro.1994.1030 8259663

[B89] Schultheiss T. Sommergruber W. Kusov Y. Gauss-Muller V. (1995). Cleavage specificity of purified recombinant hepatitis A virus 3C proteinase on natural substrates. *J. Virol.* 69 1727–1733. 7853510 10.1128/jvi.69.3.1727-1733.1995 PMC188776

[B90] Segundo D. S. Weiss M. Perez-Martín E. Koster M. J. Zhu J. Grubman M. J. (2011). Antiviral activity of bovine type III interferon against foot-and-mouth disease virus. *Virology* 413 283–292. 10.1016/j.virol.2011.02.023 21435672

[B91] Shi X. Zhang G. Wang L. Li X. Zhi Y. Wang F. (2011). The nonstructural protein 1 papain-like cysteine protease was necessary for porcine reproductive and respiratory syndrome virus nonstructural protein 1 to inhibit interferon-beta induction. *DNA Cell Biol.* 30 355–362. 10.1089/dna.2010.1188 21438756

[B92] Sim A. C. Luhur A. Tan T. M. Chow V. T. Poh C. L. (2005). RNA interference against Enterovirus 71 infection. *Virology* 341 72–79. 10.1016/j.virol.2005.06.047 16083932

[B93] Stavrou S. Feng Z. Lemon S. M. Roos R. P. (2010). Different strains of Theiler’s murine encephalomyelitis virus antagonize different sites in the type I interferon pathway. *J. Virol.* 84 9181–9189. 10.1128/JVI.00603-10 20610716 PMC2937600

[B94] Strebel K. Beck E. (1986). A second protease of foot-and-mouth disease virus. *J. Virol.* 58 893–899. 3009894 10.1128/jvi.58.3.893-899.1986 PMC252997

[B95] Sun Y. Wang Y. Shan C. Chen C. Xu P. Song M. (2012). Enterovirus 71 VPg uridylation uses a two-molecular mechanism of 3D polymerase. *J. Virol.* 86 13662–13671. 10.1128/JVI.01712-12 23055549 PMC3503026

[B96] Toyoda H. M. J. Nicklin M. G. Murray C. W. Anderson J. J. Dunn F. W. Studier F. W. (1986). A second virus-encoded proteinase involved in proteolytic processing of poliovirus polyprotein. *Cell* 45 761–770. 10.1016/0092-8674(86)90790-7 3011278

[B97] Vaccari J. P. D. R. Dietrich W. D. Keane R. W. (2014). Activation and regulation of cellular inflammasomes: gaps in our knowledge for central nervous system injury. *J. Cereb. Blood Flow Metab.* 34 369–375. 10.1038/jcbfm.2013.227 24398940 PMC3948131

[B98] Van P. V. Van E. O. Michiels T. (2001). The leader protein of Theiler’s virus inhibits immediate-early alpha/beta interferon production. *J. Virol.* 75 7811–7817. 10.1128/JVI.75.17.7811-7817.2001 11483724 PMC115023

[B99] Wang B. Xi X. Lei X. Zhang X. Cui S. Wang J. (2013). Enterovirus 71 protease [2A.sup.pro] targets MAVS to inhibit anti-viral type I interferon responses. *PLoS Pathog.* 9 :e1003231. 10.1371/journal.ppat.1003231 23555247 PMC3605153

[B100] Wang C. Sun M. Yuan X. Ji L. Jin Y. Cardona C. J. (2017). Enterovirus 71 suppresses interferon responses by blocking Janus kinase (JAK)/signal transducer and activator of transcription (STAT) signaling through inducing karyopherin-α1 degradation. *J. Biol. Chem.* 292 10262–10274. 10.1074/jbc.M116.745729 28455446 PMC5473229

[B101] Wang D. Fang L. Li K. Zhong H. Fan J. Ouyang C. (2012). Foot-and-mouth disease virus 3C protease cleaves NEMO to impair innate immune signaling. *J. Virol.* 86 9311–9322. 10.1128/JVI.00869-14 22718831 PMC3416110

[B102] Wang D. Fang L. Liu L. Zhong H. Chen Q. Luo R. (2011). Foot-and-mouth disease virus (FMDV) leader proteinase negatively regulates the porcine interferon-λ1 pathway. *Mol. Immunol.* 49 407–412. 10.1016/j.molimm.2011.09.009 21975014

[B103] Wang D. Fang L. Wei D. Zhang H. Luo R. Chen H. (2014). Hepatitis A virus 3C protease cleaves NEMO to impair induction of beta interferon. *J. Virol.* 88 10252–10258. 10.1128/JVI.00869-14 24920812 PMC4136334

[B104] Wang L. Chen S. Chang S. Lee Y. Yu C. Chen C. (2015). Enterovirus 71 proteins 2A and 3D antagonize the antiviral activity of gamma interferon via signaling attenuation. *J. Virol.* 89 7028–7037. 10.1128/JVI.00205-15 25926657 PMC4473548

[B105] Weinberg M. S. Morris K. V. (2016). Transcriptional gene silencing in humans. *Nucleic Acids Res.* 44 6505–6517. 10.1093/nar/gkw139 27060137 PMC5001580

[B106] Wu J. Chen Z. J. (2014). Innate immune sensing and signaling of cytosolic nucleic acids. *Annu. Rev. Immunol.* 32 461–488. 10.1146/annurev-immunol-032713-120156 24655297

[B107] Xia H. Wang P. Wang G. C. Yang J. Sun X. Wu W. (2015). Human Enterovirus nonstructural protein 2CATPase functions as both an RNA helicase and ATP-independent RNA chaperone. *PLoS Pathog.* 11 :e1005067. 10.1371/journal.ppat.1005067 26218680 PMC4517893

[B108] Xu C. He X. Zheng Z. Zhang Z. Wei C. Guan K. (2014). Downregulation of MicroRNA miR-526a by Enterovirus inhibits RIG-I-dependent innate immune response. *J. Virol.* 88 11356–11368. 10.1128/JVI.01400-14 25056901 PMC4178780

[B109] Xue Q. Liu H. Zhu Z. Yang F. Ma L. Cai X. (2018). Seneca Valley Virus 3Cpro abrogates the IRF3- and IRF7-mediated innate immune response by degrading IRF3 and IRF7. *Virology* 518 1–7. 10.1016/j.virol.2018.01.028 29427864

[B110] Yang Y. Liang Y. Qu L. Chen Z. Yi M. Li K. (2007). Disruption of innate immunity due to mitochondrial targeting of a picornaviral protease precursor. *Proc. Natl. Acad. Sci. U.S.A.* 104 7253–7258. 10.1073/pnas.0611506104 17438296 PMC1855380

[B111] Zhao T. Yang L. Sun Q. Arguello M. Ballard D. W. Hiscott J. (2007). The NEMO adaptor bridges the nuclear factor-kappaB and interferon regulatory factor signaling pathways. *Nat. Immunol.* 8 592–600. 10.1038/ni1465 17468758

[B112] Zheng Z. Li H. Zhang Z. Meng J. Mao D. Bai B. (2011). Enterovirus 71 2C protein inhibits TNF-α-mediated activation of NF-κB by suppressing IκB kinase β phosphorylation. *J. Immunol.* 187 2202–2212. 10.4049/jimmunol.1100285 21810613

[B113] Zinzula L. Tramontano E. (2013). Strategies of highly pathogenic RNA viruses to block dsRNA detection by RIG-I-like receptors: hide, mask, hit. *Antiviral Res.* 100 615–635. 10.1016/j.antiviral.2013.10.002 24129118 PMC7113674

